# Electrophysiological and Structural Remodeling in Heart Failure Modulate Arrhythmogenesis. 1D Simulation Study

**DOI:** 10.1371/journal.pone.0106602

**Published:** 2014-09-05

**Authors:** Juan F. Gomez, Karen Cardona, Lucia Romero, Jose M. Ferrero, Beatriz Trenor

**Affiliations:** Instituto de Investigación en Ingeniería Biomédica, Universitat Politècnica de València, Valencia, Spain; Gent University, Belgium

## Abstract

**Background:**

Heart failure is a final common pathway or descriptor for various cardiac pathologies. It is associated with sudden cardiac death, which is frequently caused by ventricular arrhythmias. Electrophysiological remodeling, intercellular uncoupling, fibrosis and autonomic imbalance have been identified as major arrhythmogenic factors in heart failure etiology and progression.

**Objective:**

In this study we investigate *in silico* the role of electrophysiological and structural heart failure remodeling on the modulation of key elements of the arrhythmogenic substrate, i.e., electrophysiological gradients and abnormal impulse propagation.

**Methods:**

Two different mathematical models of the human ventricular action potential were used to formulate models of the failing ventricular myocyte. This provided the basis for simulations of the electrical activity within a transmural ventricular strand. Our main goal was to elucidate the roles of electrophysiological and structural remodeling in setting the stage for malignant life-threatening arrhythmias.

**Results:**

Simulation results illustrate how the presence of M cells and heterogeneous electrophysiological remodeling in the human failing ventricle modulate the dispersion of action potential duration and repolarization time. Specifically, selective heterogeneous remodeling of expression levels for the Na^+^/Ca^2+^ exchanger and SERCA pump decrease these heterogeneities. In contrast, fibroblast proliferation and cellular uncoupling both strongly increase repolarization heterogeneities. Conduction velocity and the safety factor for conduction are also reduced by the progressive structural remodeling during heart failure.

**Conclusion:**

An extensive literature now establishes that in human ventricle, as heart failure progresses, gradients for repolarization are changed significantly by protein specific electrophysiological remodeling (either homogeneous or heterogeneous). Our simulations illustrate and provide new insights into this. Furthermore, enhanced fibrosis in failing hearts, as well as reduced intercellular coupling, combine to increase electrophysiological gradients and reduce electrical propagation. In combination these changes set the stage for arrhythmias.

## Introduction

Heart failure (HF) is a clinical syndrome characterized by the marked and progressive inability of the ventricles to fill and generate adequate cardiac output to meet the demands of cellular metabolism that may have significant variability in its etiology [1]. HF is a final common pathway of a multitude of cardiac pathologies. Congenital cardiac abnormalities, valve disease, hypertension, dilated cardiomyopathy, hypertrophic cardiomyopathy, arrhythmogenic right ventricular cardiomyopathy, and ischemic damage can all result eventually in HF. The primary electrophysiological changes and the mechanisms for arrhythmogenesis associated with HF depend on its etiology [2]. Thus, HF is not characterized by a single set of electrophysiological changes. However, there are a number of consistent findings thought to be important for arrhythmogenesis. These include: ion channel remodeling, enhanced cellular uncoupling, altered calcium homeostasis, extracellular matrix changes, and increased sympathetic nerve activity [2].

Marked ion channel remodeling in failing human hearts has been described in several experimental studies [3]–[11]. However detailed studies focusing heterogeneous remodeling within the ventricular free wall are lacking [12]–[16]. This study was undertaken to evaluate how transmural heterogeneous remodeling of selected ion channels in failing hearts modulates electrophysiological dispersion in the ventricular myocardium, which is known to set the stage for malignant arrhythmias [17]–[19].

Furthermore, structural remodeling and cellular uncoupling [30]–[34] in failing hearts have long been known to contribute to arrhythmogenesis [20]–[22]. However, proliferation of fibroblasts and their possible electrical interaction with cardiac myocytes [23]–[29] has only recently emerged as a plausible pro-arrhythmic factor. These factors are known to alter electrical patterns of propagation and dispersion.

Enhanced dispersion of repolarization in the failing heart, due to a heterogeneous distribution of action potential duration (APD) through the myocardial wall, is thought to be an important contributor to arrhythmia [17]–[19], [30]. However, a recent experimental study by Glukhov et al. [31] reported that transmural APD gradients were decreased in the failing ventricle compared with non-failing hearts.

Computational and theoretical analyses may provide useful insights into the mechanisms responsible for this controversy. Mathematical models of disease-specific AP in human heart are powerful tools for improvement of basic understanding of the ionic mechanism(s) of dysfunction in disease, such as arrhythmias and HF. Furthermore, anatomically detailed multiscale models (from cell to organ dimensions) often can provide complementary approach to clinical and physiological measurements with the common goal of helping to optimize medical devices and pharmacological treatments (see [32], [33] for review).

In the present study, the electrophysiological activity of a transmural one dimensional wedge was simulated using well-justified modifications on recent mathematical models of human ventricular AP [34], [35] in an attempt to replicate the experimentally reported human HF phenotypes. The influence of electrophysiological and structural remodeling on transmural dispersion of repolarization, Ca^2+^ transients, and electrical conduction properties were the foci of this work. New insights include: the effects of (i) transmural heterogeneous remodeling of SERCA pump and Na^+^/Ca^2+^, (ii) the presence of M cells, and (iii) the presence of fibrosis and cellular uncoupling, on repolarization and Ca^2+^ transient gradients.

## Methods

### Cellular models

Simulations of the electrophysiological activity of endocardial and epicardial human ventricular myocytes were carried out using one of the latest human ventricular AP models, developed by Grandi et al. (GPB model) [34], and characterized by a thorough description of intracellular calcium handling. The O'Hara et al. ventricular AP model (ORd) [35] was also employed to simulate the electrical activity of epicardial, endocardial, and M cells, defined as midmyocardial cells with prolonged APD [36]. Indeed, GPB model does not include a formulation for M cells. ORd model is based on experimental data taken from 140 non-diseased human hearts. It can reproduce the electrophysiological behavior of all three types of human ventricular myocytes, in accordance with experimental observations.

### Model of ventricular fibroblast

To simulate the electrical activity of fibroblasts, the formulation of MacCannell et al. [37] was used, which is based on experimental data from adult rat ventricles [38]. Specifically, the active fibroblast model includes a time and voltage-dependent potassium current (I_K_), a voltage-dependent inward-rectifying K^+^ current (I_K1_), as well as a Na^+^/K^+^ pump (I_NaK_), and a background sodium current (I_b,Na_), with a membrane capacitance of 6.3 pF. The uncoupled resting membrane potential of the active ventricular fibroblast is −49.6 mV.

### Homogeneous electrophysiological remodeling in heart failure

To simulate the electrical activity of human ventricular failing myocytes, GPB and ORd models were modified. Quite extensive parameters were changed (based on experimental data from human when it was possible) to describe the hallmark characteristics of failing cardiac tissues and cells, such as AP prolongation and alteration of Ca^2+^ handling [39], [40]. A detailed description of these HF models was previously published by our group [41], [42]. The entire set of currents and parameter modifications (applied homogeneously in all types of cells) are summarized in Table 1. As mentioned, ORd model includes an electrical description for M cells so that heterogeneous HF remodeling in endocardial, M, and epicardial cells was possible to be defined.

**Table 1:** Heart failure ionic remodeling.

Ionic Parameter modified	% in the HF model compared to the normal model		Experimental conditions
	GPB model	ORd model	
**I_NaL_**	200%	180%	Isolated cardiomyocytes from LV mid-myocardium of failing dog hearts [88] Whole cell voltage clamp (room temperature) [3]
**τ_hL_**	200%	180%	Isolated cardiomyocytes from LV mid-myocardium of failing dog hearts [88] Whole cell voltage clamp (room temperature) [3]
**I_to_**	40%	40%	Isolated cardiomyocytes from LV mid-myocardium of failing human hearts. Whole cell voltage clamp (room temperature) [4]
**I_K1_**	68%	68%	Review article. Several species [5]
**I_NaK_**	90%	70%	Measurements of human myocardial [7] Na,K-ATPase concentration in failing hearts [5]Review article. Several species [6]
**I_Nab_**	0	100%	Simulation of human HF [89]
**I_Cab_**	153%		Simulation of human HF [89]
**I_NCX_**	175%	175%	Simulation of human HF [90]
**J_SERCA_**	50%	50%	Isolated cardiomyocytes from LV of failing human hearts. Measurements of Ca^2+^uptake rates by the SR (37°C) [8]
**I_leak_**	300%	130%	Review article [9]
**EC_50SR_**	89%		Review article [10]Isolated cardiomyocytes from LV of failing rabbit hearts. Measurements of RyR sensitivity to SR Ca^2+^ [91]
**CaMKa**		150%	Review article [10]. Transgenic mice [11]
**J_rel,NP,∞ Ca_^2+^_sensitivity_**		80%	Review article [92]

Changes in original Grandi et al. (GPB) [34] and O'Hara et al. (ORd) [35] models to simulate heart failure (HF) phenotype. The modified parameters are: the late Na^+^ current (I_NaL_), the time constant of inactivation of the I_NaL_ (**τ**_hL_), the transient outward K^+^ current (I_to_), the inward rectifier K^+^ current (I_K1_), the Na^+^/K^+^ pump current (I_NaK_), the background Ca^2+^ current (I_Cab_), the Na^+^/Ca^2+^ exchanger (I_NCX_), the sarcoplasmic reticulum (SR) Ca^2+^ pump (J_SERCA_), the SR Ca^2+^ leak current (I_leak_), the Ca^2+^ sensitivity of SR Ca^2+^ fluxes (EC_50SR_), the Ca^+2^calmodulin-dependent protein kinase II, and the non-phosphorylated Ca^2+^ release, via ryanodine receptors (J_rel,NP,∞_).

### Heterogeneous electrophysiological remodeling in heart failure

Experimental studies describing the functional features and changes in expression levels of ion channels across the ventricular free wall in the failing human heart are scarce [12]–[16]. Furthermore, extrapolating protein expression levels to channel functional activity is not trivial. Thus, on the basis of the limited literature, we proposed a heterogeneous model of HF based on our previous work [43], where specific parameters were differently altered in epicardial, endocardial, and M cells. Specifically, the activity of the Na^+^/Ca^2+^ exchanger (I_NCX_), which shows a significant upregulation in failing myocytes, was increased 2-fold in epicardial cells and 1.6-fold in M and endocardial cells [15], [43], as shown in Table 2. Heterogeneous downregulation of the SERCA pump (I_SERCA_) has also been described experimentally [16], and was applied in our simulations as follows: a 55% reduction in endocardial cells, a 25% reduction in epicardial cells and a 40% reduction in M cells (see Table 2). These changes which strongly modulate Ca^2+^ transient, were considered as a starting point for heterogeneous remodeling. Transmural heterogeneous remodeling of K^+^ currents has also been reported in the human failing ventricular wall, mainly the transient outward K^+^ current (I_to_) [20], [44]. Heterogeneous remodeling of I_to_ would surely have an impact on early repolarization; however this is out of the scope of the present work.

**Table 2:** Heart failure heterogeneous transmural ionic remodeling.

Remodeled Current	% in the HF model compared to the normal GPB/ORd models			Experimental conditions
	Epi	M	Endo	
**I_NCX_**	200%	160%	160%	Simulation of human HF [89]. The functional activity of the Na^+^-Ca^2+^ exchanger was determined by measuring the Na^+^-dependent Ca^2+^ uptake into membrane vesicles prepared from human left ventricular samples[93]. Isolated cardiomyocytes from failing canine hearts. Data of transmural NCX mRNA, protein levels, and current [15]. Cellular and transmural 1D simulations of HF using ventricular AP canine model [94].
**I_SERCA_**	75%	60%	45%	Isolated cardiomyocytes from LV of failing human hearts. Measurements of Ca^2+^uptake rates by the SR (37°C) [8]. Endocardial strip preparations from human failing hearts. Measurements of Ca^2+^uptake in myocardial homogenates (37°C) [95]. LV from human failing hearts. Measurements of Ca^2+^uptake [96]. Explanted human failing hearts. Data of transmural protein levels of SERCA2a [16].

Changes in original Grandi et al. (GPB) [34] and O'Hara et al. (ORd) [35] models to simulate heart failure (HF) heterogeneous remodeling. The heterogeneously modified parameters are: the Na^+^/Ca^2+^ exchanger (I_NCX_), the sarcoplasmic reticulum (SR) Ca^2+^ pump (J_SERCA_). Different percentages of these currents with respect to the control value were used in epicardium, endocardium and M-cells (in the case of the ORd model).

### Computational methods

To carry out these simulations in strand models of human ventricle we used ELVIRA software [45]. This is based on a pseudo-adaptive finite element method in space and time to solve anisotropic reaction–diffusion equations with highly nonlinear reactive terms. The reaction-diffusion equation (1) governs AP propagation through the multicellular strand. The strand was spatially discretized into linear elements (Δx = 0.01 cm) delimited by two nodes, one in each of the element ends, where the ionic model is solved. The temporal resolution was fixed to t = 0.002 ms to ensure convergence. The scheme accounts for the anisotropy of the media and incorporates an adaptive time step algorithm to integrate the stiff reactive term associated with the ionic currents. The resolution of the monodomain equation (1) is based on the technique of operator splitting. 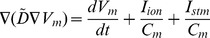
(1)

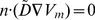
(2)


Where V_m_ is membrane potential in (V), 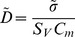 in (m^2^/s) is the diffusion conductivity tensor,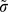 is the conductivity tensor in (S.m), 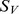 is the surface to volume ratio of the cell in (m^−1^), 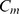 in (F) is the membrane capacity, 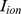 in (A) is the ionic total current, and 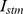 in (A) is the stimulus current. Equation (1) has boundary conditions (2). Further information can be found in [45].

The ionic model that is solved in each node of the discretized domain changes depending on the described cell (myocyte [34], [35] or fibroblast [37]). Temporal equations are computed using a semi-implicit numerical method (implicit for the parabolic equation and explicit for ionic models at each node).

### Human ventricular transmural strand models

To simulate the electrical activity of a transmural wedge preparation, a heterogeneous one dimensional strand composed of 165 cells was considered as in [35], [46], [47]. This corresponds to a thickness of 1.65 cm, which is within the range of 1 to 2 cm reported for the width of the human left ventricular wall [48]. The strand was divided into 82 endocardial cells and 83 epicardial cells, each myocyte being driven by GPB formulation [34]. The diffusion tensor (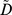) in normal conditions (NC) for myocyte cells was set to D_M_ = D_xx_ = 0.0006 cm/ms resulting in a conduction velocity (CV) of 50 cm/s as in experimental measurements of transmural conduction [49]. The intercellular uncoupling observed in failing hearts was modeled by a two-fold decrease in diffusion coefficient, i.e. intercellular conductivity was halved (D_M_ = 0.0003 cm/ms), in accordance with experimental measurements in the human failing heart reporting a 50% reduction in connexin43 protein levels [50]. To test other degrees of intercellular uncoupling, mild uncoupling (D_M_ = 0.00045 cm/ms) and severe uncoupling (D_M_ = 0.00025 cm/ms) were also considered. The effect of fibroblasts within the strand was simulated by (i) randomly distributing fibroblasts (diffuse fibrosis) or (ii) considering small localized clusters (patchy fibrosis) [51], [52]. Diffuse fibrosis is defined as small deposits of fibrous tissue. Fibroblasts were thus randomly distributed within the strand so that some nodes were assigned to the fibroblast ionic model and the rest of the nodes to the myocyte ionic model. As in [53], a fibrotic content of 10% or 20% corresponds to the percentage of nodes assigned to the fibroblast ionic model. For each fibrotic content 11 random configurations were simulated (see Figures S1 and S2 in File S1). Patchy fibrosis, i.e. larger clusters of fibrous tissue, was simulated with clusters of 25 interconnected fibroblasts, introduced within the ventricular strand. Fibroblasts were electrotonically coupled to surrounding cells (myocytes or other fibrotic cells) by considering a lower conductivity tensor with respect to conductivity tensor between myocytes. The diffusion coefficient in fibroblast elements was decreased three-fold with respect to myocytes, based on experimental data [27]. To test the effects of the heterogeneous electrophysiological remodeling characteristic of HF, multicellular strands containing also M cells were considered. For this purpose, one dimensional simulations were performed using a heterogeneous multicellular strand, which resembles some functional features of a ventricular transmural wedge preparation, as described in O'Hara et al. [35]. This strand was composed by 60 endocardial, 45 M, and 65 epicardial cells governed by ORd formulation. Stimuli were applied at the extreme endocardial end in all configurations with a basic cycle length (BCL) of 1000 ms. Parameters were measured for the last stimulation, after steady state was reached, i.e. after 200 s and 750 s in the cellular strands using GPB and ORd models, respectively.

### Parameter definition

APD at 90% of repolarization (APD_90_) was computed in myocytes of the strand, then APD dispersion was calculated as the difference between the maximum and minimum APD_90_ along the strand. Border effects were avoided by eliminating 15 cells in each end of the strand for the computation of dispersion. Repolarization time (RT) for a cell was measured by adding APD_90_ of this cell to the time needed by the wavefront to reach the cell. Transmural dispersion of repolarization (TDR) was then computed as the difference between the maximum and minimum RT along the multicellular strand. Ca^2+^ transient duration was measured as the time from the upstroke to 80% of recovery (CaTD_80_) as in [54], and the voltage-calcium delay was defined as the delay between the upstrokes of AP and Ca^2+^ transient (AP-Ca delay) [54]. CaTD_80_ and AP-Ca delay dispersions were computed as the difference between the maximum and minimum CaTD_80_ and AP-Ca delay, respectively, along the multicellular strand.

The effective refractory period (ERP) was measured as the minimum time between two consecutive stimuli that resulted in propagation of the AP wave across the strand. Conduction velocity (CV) was measured between the 15^th^ and 150^th^ myocytes (to avoid edge effects), as the distance between cells divided by the difference between the times of maximum depolarization upstroke. The safety factor (SF) for conduction is a quantitative parameter based on the source-sink relationship of electric charge between adjacent cardiac cells. The value of the SF indicates the success or failure in AP propagation. Several definitions of the SF have been published [55]–[57], and the formulation proposed by Romero and coworkers [58] (SF_m_), described in equation (3), was chosen because of its computational advantages. This formulation of the SF was proposed by Shaw and Rudy [56], however, Romero et al. [58] changed the definition of the integration domain, optimizing its calculation and saving computational resources.
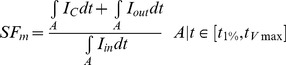
(3)where I_c_ stands for the capacitive current, I_in_ is the axial current that enters the cell, I_out_ is the axial current that leaves the cell, t_1%_ in the instant when membrane potential reaches 1% of its maximum and t_Vmax_ is the instant of maximal V_m_.

### Statistical analysis

SPSS Statistics 17.0 software (IBM SPSS Statistics) was used for student t-test. Values are expressed as mean±SD. p<0.05 was considered significant.

## Results

### Effects of electrophysiological remodeling on transmural repolarization gradients

**Figure 1:**
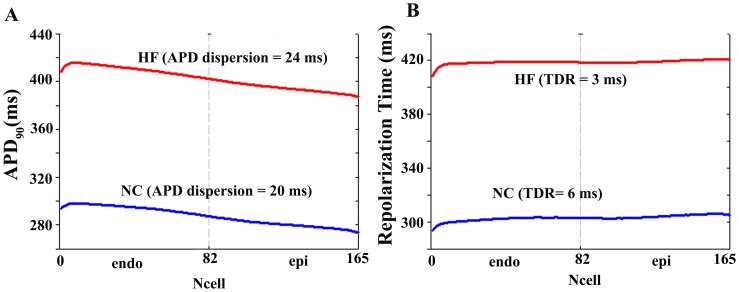
Action potential duration and repolarization time in normal and failing conditions. A. Action potential duration (APD_90_) measured along a transmural ventricular strand, composed by 82 endocardial and 83 epicardial cells, in normal conditions (NC, blue line) and in heart failure conditions (HF, red line). APD dispersion is indicated in ms for each curve. B. Repolarization time calculated along the ventricular strand in normal (blue line) and failing (red line) conditions. Transmural dispersion of repolarization (TDR) was measured in ms. Simulations were conducted using Grandi et al. (GPB) action potential model [34].

To assess the influence of HF induced electrophysiological remodeling on repolarization gradients, detailed simulations of the action potential conduction and repolarization in human ventricular transmural wedge preparations were carried out using our modified GPB and ORd AP models. Figure 1 shows the results obtained in a wedge composed by endocardial and epicardial myocytes using GPB model. As depicted in panel A, APD_90_ is longer in the failing endocardial and epicardial myocytes (red line) than in normal conditions (blue line) at a stimulation rate of 1 Hz. Note also that APD dispersion is increased in HF to 24 ms versus 20 ms in control conditions. In addition, as shown in panel B, repolarization time along the strand is delayed in failing myocytes (red line). Transmural dispersion of repolarization was slightly decreased in HF with respect to control conditions.

**Figure 2:**
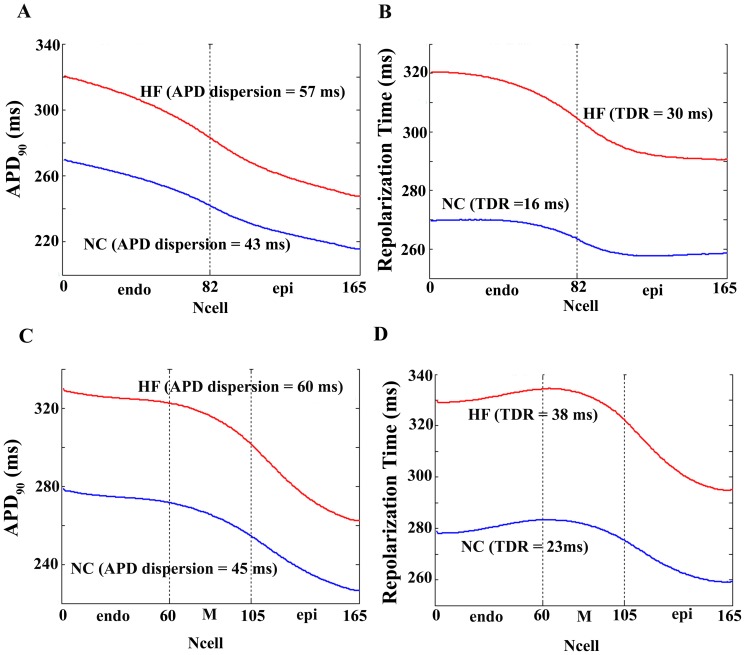
Action potential duration and repolarization time in normal and failing conditions including M cells influence. Panels A and C. Action potential duration (APD_90_) measured along a transmural ventricular strand, in normal conditions (NC, blue line) and in heart failure conditions (HF, red line). APD dispersion is indicated in ms for each curve. Panels B and D. Repolarization time calculated along the ventricular strand in normal (blue line) and failing (red line) conditions. Transmural dispersion of repolarization (TDR) was measured in ms. The ventricular strands are composed by 82 endocardial and 83 epicardial cells (panels A and B) and by 60 endocardial, 45 M-, and 65 epicardial cells (panels C and D). All simulations were conducted using O'Hara et al. (ORd) action potential model [35].

Similar simulations were conducted using the modified ORd model to assess whether this pattern of results were model-dependent. From Figure 2 (panels A and B) it is clear that for ORd model values of APD and repolarization time are also lengthened in HF compared with NC. As expected, some differences with respect to GPB results were observed. The increase in APD dispersion was more pronounced in HF when ORd model was used. TDR increased in HF whereas the opposite result was obtained with the GPB model. Significant differences in the formulation of repolarizing currents of ORd and GPB models, as described with detail in [59] are likely responsible for these differences. Especially, the fact that ORd model considers transmural differences in 11 ionic currents, while GPB model distinguishes only between I_to_ in epicardial and endocardial myocytes, might be responsible for the different behaviors of the models in terms of APD transmural gradients and TDR.

To analyze the influence of M cells on repolarization gradients in the failing tissue, a transmural wedge in which M cells were also implemented. In this analysis only ORd model was used, as GPB model does not include any AP formulation for this type of cells. APD dispersion and TDR along a transmural ventricular strand comprising M cells are shown in Figure 2 (panels C and D). The presence of M cells prolongs APD throughout the fiber and increases its dispersion (panel C) in both control and HF. Repolarization time is also increased at each fixed location in the strand and TDR also increased (panel D). However, the relative increase of APD dispersion and TDR in HF with respect to NC, in the presence of M cells is very similar to these changes in their absence. Thus, as expected from their intrinsic properties, M cells increase global repolarization gradients but do not increase the relative changes due to HF electrophysiological remodeling (with respect to control).

### Effects of heterogeneous electrophysiological remodeling on transmural repolarization gradients

**Figure 3:**
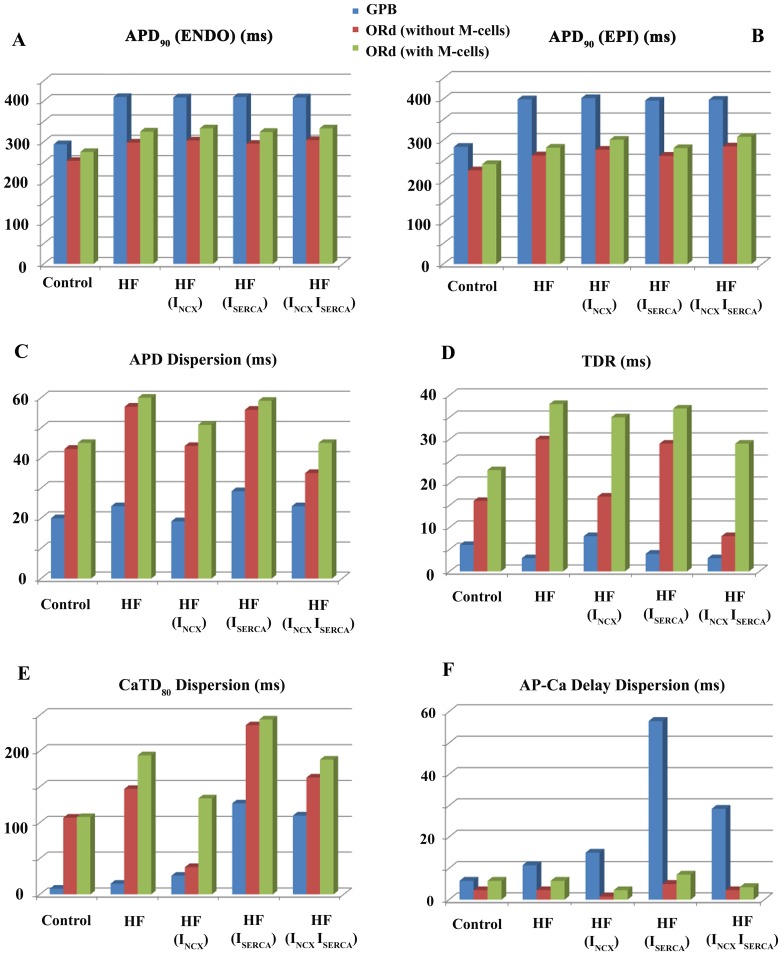
Biomarkers in normal, homogeneous and heterogeneous remodeling failing conditions with GPB, ORd (without M cells), and ORd (with M cells) models. Electrophysiological properties measured in a one dimensional transmural ventricular strand under different pathological conditions. The ventricular strands were composed by 82 endocardial and 83 epicardial cells. All simulations were conducted using Grandi et al. (GPB) action potential model [34] (blue bars), O'Hara et al. [35] model (ORd) without M-cells (red bars) and ORd model with M cells (green bars). The cases considered were: normal conditions (Control), electrical homogeneous heart failure remodeling (HF), electrical heterogeneous heart failure remodeling on I_NCX_ (HF I_NCX_), electrical heterogeneous heart failure remodeling on I_SERCA_ (HF I_SERCA_), and electrical heterogeneous heart failure remodeling on I_NCX_ and I_SERCA_ simultaneously (HF I_NCX_I_SERCA_). The mean action potential duration (APD) along the endocardial (panel A) and epicardial (panel B) strand was measured at 90% repolarization. Dispersion in APD (panel C) was measured along the entire strand as the difference between maximal and minimal APD, avoiding first and last fifteen cells. Transmural dispersion of repolarization (TDR) (panel D) was measured by adding the APD to the time of depolarization of each cell. The calcium transient duration (CaTD_80_) (panel E) was measured from the upstroke to 80% of calcium transient amplitude, dispersion was assessed as in APD. AP-Ca delay (panel F) was measured as the difference between the upstroke in AP and in calcium transient along the entire strand, dispersion was also evaluated as in APD (see Methods section for details).

Several experimental studies [12]–[16] have reported differences in the remodeling across the transmural ventricular free wall in failing hearts. To analyze how heterogeneous remodeling can affect APD_90_ and corresponding repolarization time gradients, simulations were performed using HF models with spatially heterogeneous remodeling across the ventricular strand, as described in the Methods section. Figure 3 shows the simulation results for GPB model in endocardium and epicardium (blue bars), for ORd model in endocardium and epicardium (red bars), and for ORd model also including M cells (green bars). In the case of GPB model, heterogeneous remodeling of I_NCX_ decreased APD dispersion to 19 ms versus 24 ms corresponding to homogeneous HF remodeling and 20 ms corresponding to normal conditions (panel C Figure 3). Similarly, using ORd model, heterogeneous remodeling of I_NCX_ and/or I_SERCA_ decreased APD dispersion with respect to homogeneous HF remodeling, regardless of the absence (red bars) or presence (green bars) of M cells. TDR (Figure 3 panel D) was also modulated by heterogeneous remodeling and was decreased with respect to homogeneous remodeling when ORd model was used. In general, in the presence of M cells, the values of APD dispersion and TDR are higher, and heterogeneous HF remodeling does not bring these values lower than control values.

### Effects of heterogeneous electrophysiological remodeling on transmural dispersion of Ca^2+^ transients

**Figure 4:**
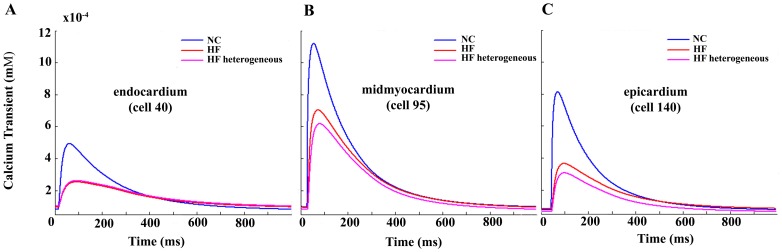
Calcium transient representation under normal and failing conditions. Ca^2+^ transient representation in an endocardial cell (#40) (panel A), in a midmyocardial cell (#95) (panel B), and in an epicardial cell (#140) (panel C) of a heterogeneous ventricular strand for normal conditions (NC blue lines), homogeneous failing conditions (HF red lines), and heterogeneous heart failure (HF) remodeling (magenta lines). Simulations were performed using O'Hara et al. model [35].

**Figure 5:**
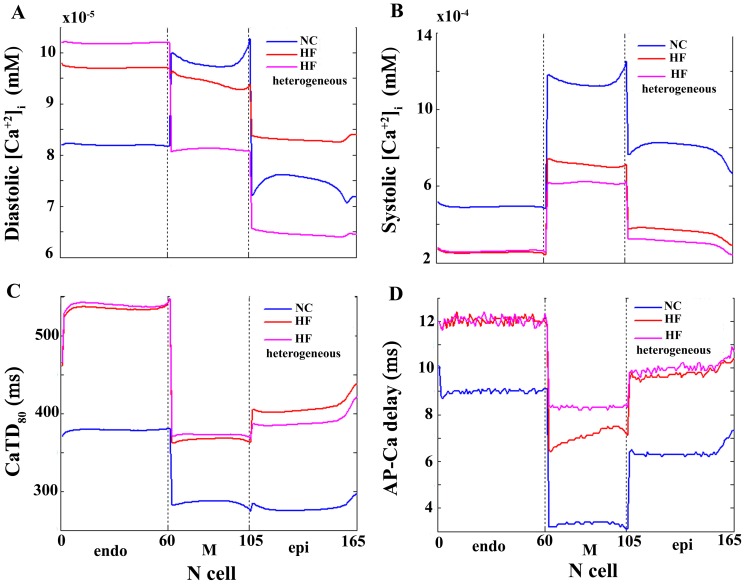
Calcium transient dynamics under normal and failing conditions. Diastolic and systolic [Ca^2+^]_i_ levels (panels A and B, respectively), Ca^2+^ transient duration (CaTD_80_) (panel C), and delay between AP and Ca^2+^ transient upstrokes (AP-Ca delay) (panel D) along a transmural ventricular strand under normal conditions (NC blue lines), homogeneous heart failure (HF) remodeling (red lines), and heterogeneous HF remodeling conditions (pink lines). Simulations were performed using O'Hara et al. model [35].

Because the heterogeneous remodeling that is the focus of the paper is limited to I_NCX_ and I_SERCA_, which strongly affect Ca^2+^ transients, we also analyzed Ca^2+^ homeostasis. Figure 4 depicts Ca^2+^ transients in three different myocytes phenotypes within the transmural strand under normal conditions (blue), and for homogeneous (red) and heterogeneous (pink) HF remodeling. As observed experimentally in HF, the amplitude of Ca^2+^ transients is decreased, and the rise and decay rates are slowed. Figure 5 depicts the values of diastolic Ca^2+^ in panel A, systolic Ca^2+^ in panel B, Ca^2+^ duration (CaTD_80_) in panel C, and the delay between AP and Ca^2+^ upstrokes (AP-Ca delay) in panel D, along the transmural strand. Significant differences in these magnitudes are observed along the ventricular strand in normal conditions (blue lines) and also after HF remodeling (red lines for homogeneous HF and pink lines for heterogeneous HF). The dispersion of CaTD_80_ and AP-Ca delay were calculated for the different AP models (panels E and F in Figure 3). HF increased CaTD_80_ dispersion in both models, which was strongly modulated by heterogeneous remodeling of I_NCX_ and I_SERCA_. AP-Ca delay dispersion was increased or unaltered in HF using GPB or ORd models, respectively. Again, heterogeneous remodeling modulated AP-Ca delay, especially when GPB model was used.

Heterogeneous remodeling in failing hearts has been considered an important contributor to the HF phenotype and likely a major factor for modulation of transmural repolarization and Ca^2+^ transient gradients, as illustrated in our simulations (see Figure 3). The presence of M-cells also contributes to this modulation, by maintaining higher gradients. However, there is limited electrophysiological data from the human heart, and further experimental studies, similar to [54], are needed to fully determine how heterogeneous remodeling modulates APD gradients, TDR and Ca^2+^ transients changes. Specifically, measurements of AP and Ca^2+^ transients transmural gradients and their relation to protein expression levels or activity of the SERCA pump and I_NCX_ in a transmural wedge of the failing heart would be very valuable.

### Effects of structural remodeling on repolarization gradients and impulse conduction

**Figure 6:**
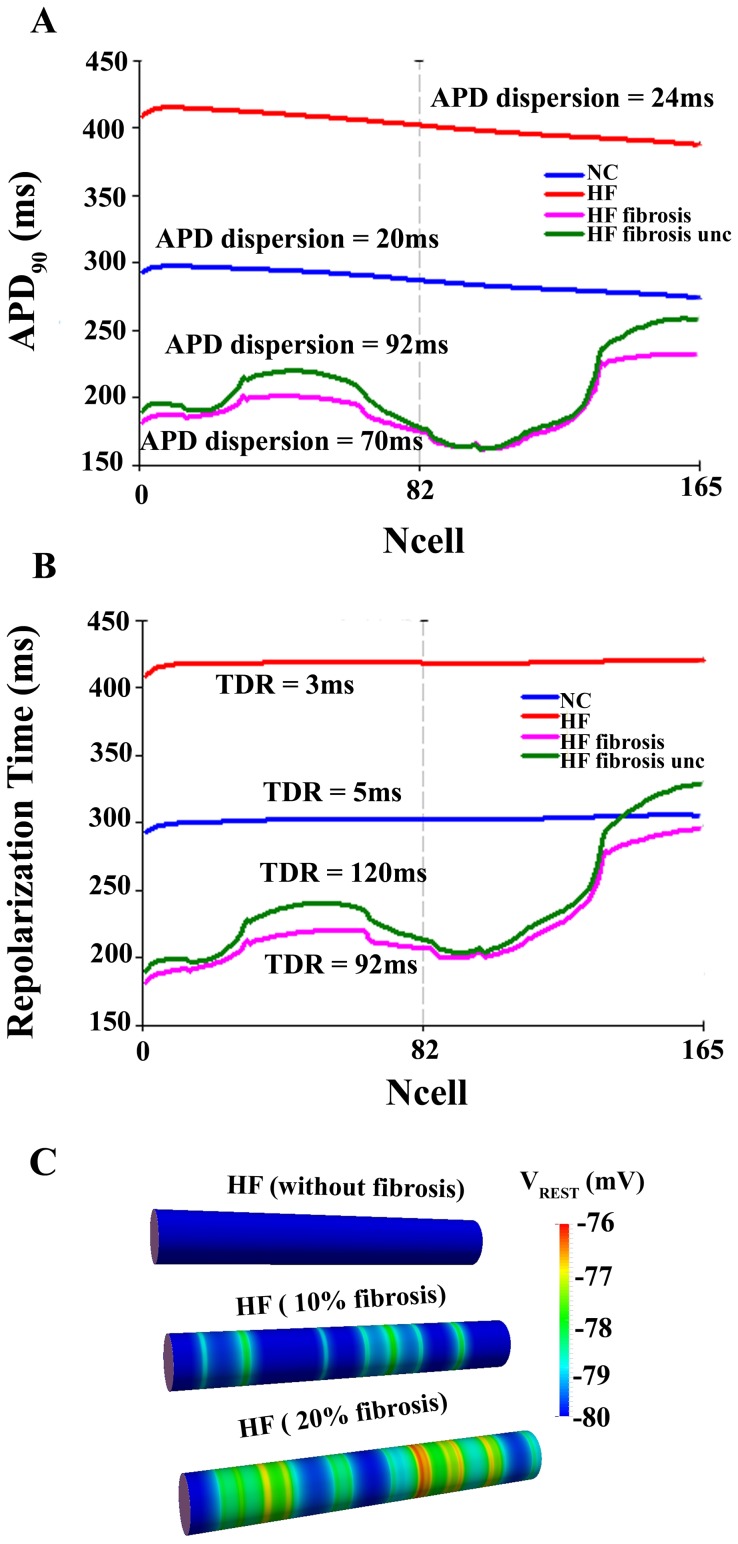
Action potential duration and repolarization time in normal and failing conditions with structural remodeling. Action potential duration (APD_90_) (panel A) and repolarization time (panel B) measured along transmural ventricular strands, in normal conditions (NC, blue lines), in heart failure electrophysiological remodeling conditions (HF, red lines), in heart failure electrophysiological remodeling conditions with fibrosis 10% (HF fibrosis, pink lines), and in heart failure electrophysiological remodeling conditions with fibrosis 10% and intercellular uncoupling (HF fibrosis unc, green lines). APD dispersion and transmural dispersion of repolarization (TDR) were measured in ms for each curve. The ventricular strands were composed by 82 endocardial and 83 epicardial cells. All simulations were conducted using Grandi et al. (GPB) action potential model [34]. Panel C shows in a color code the resting potential (V_rest_) along the failing ventricular strand after achieving steady state conditions in the absence of fibrosis, 10% fibrosis, and 20% fibrosis.

The effect of fibrosis within the strand was evaluated in a transmural ventricular wedge composed by endocardial and epicardial cells. GPB model was used because the effects of electrophysiological remodeling in repolarization gradients were less pronounced than in ORd model and we were interested in evaluating whether structural remodeling could significantly increase these gradients and/or alter action potential conduction. When clusters of fibroblasts were inserted randomly in the strand, marked differences in APD and repolarization time resulted, as shown in Figure 6. When fibrosis was present in the failing strand (10% of fibrotic content), APD dispersion went up to 70 ms, compared to 24 ms with HF electrophysiological remodeling. When intercellular uncoupling was also introduced, the APD dispersion increased even more to 92 ms.

**Figure 7:**
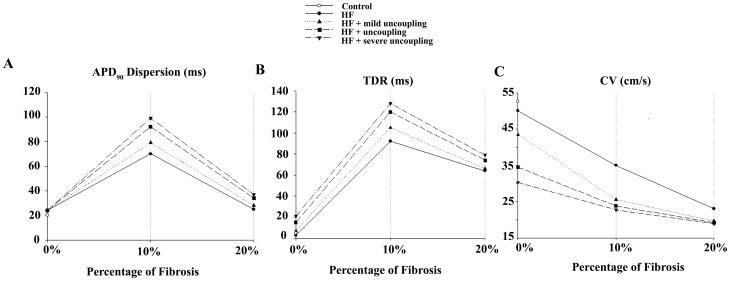
Biomarkers in normal and failing conditions adding structural remodeling with GPB. Electrophysiological properties measured in a one dimensional transmural ventricular strand under different pathological conditions. The ventricular strands were composed by 82 endocardial and 83 epicardial cells. All simulations were conducted using Grandi et al. (GPB) action potential model [34]. The cases considered were: normal conditions (NC) (white circle), electrical heart failure remodeling (HF) (solid line), electrical heart failure remodeling and mild uncoupling (dotted line), normal uncoupling (dashed line) and severe uncoupling (dotted-dashed line). Different degrees of fibrosis were considered: 10% and 20%. Action potential duration (APD) dispersion (panel A), transmural dispersion of repolarization (TDR) (panel B), and conduction velocity (CV) (panel C) along the whole strand were measured (see Methods section for details).

A similar pattern of change could be observed in TDR (see Figure 6 panel B). Transmural dispersion of repolarization was also significantly enhanced when the fibrotic content was 10%, as shown in Figure 6 panel B and even more when intercellular uncoupling was considered. Thus, inserted fibrosis and intercellular uncoupling increased TDR and APD dispersion. Nevertheless, when the fibrotic content was further enhanced to 20%, APD dispersion and TDR were decreased with respect to 10% fibrosis, showing a biphasic behavior with fibrotic content, as shown in Figure 7A and 7B. This tendency was maintained for different uncoupling degrees. The values of APD dispersion and TDR shown correspond to one of the random configurations (configuration 1) for each fibrotic content (10% and 20%). Additional random configurations were simulated and the results are shown in the Supporting Information (see Tables S1 and S2 in File S1 for normal intercellular coupling and Tables S3, S4, S5, and S6 in File S1 for the different degrees of intercellular uncoupling and fibrosis). A statistical analysis was also performed and APD dispersion and TDR mean values were lower in the case of 20% of fibrotic content with respect to 10% fibrosis, regardless of the degree of cellular uncoupling (see Tables S1, S2, S3, S4, S5, and S6 in File S1 and Figures S3, S4, S5, S6, S7, S8, S9, and S10 in File S1, however only in the case of APD dispersion this difference was statistically different (p<0.05).

The excitability and conduction properties were also analyzed in the presence of HF electrophysiological remodeling, fibrosis and/or intercellular uncoupling. Figure 6 panel C shows the color-coded resting membrane potential (V_rest_) in each cell of the unidimensional strand in HF without fibrosis, in HF with 10% fibrosis, and in HF with 20% fibrosis after a period of stabilization. As expected, V_rest_ increases with the fibrotic content. Figure 7 panel C shows that HF alone (filled circle with 0% of fibrosis) did not alter significantly CV with respect to control (white circle) but did increase the effective refractory period with respect to control (444 ms vs. 320 ms; results not shown). However, when fibrosis and intercellular uncoupling were considered, separately or concomitantly, the decrease of CV was more pronounced and synergistic. With regard to ERP, uncoupling alone had no significant effect, whereas fibrosis reduced it significantly (results not shown).

**Figure 8:**
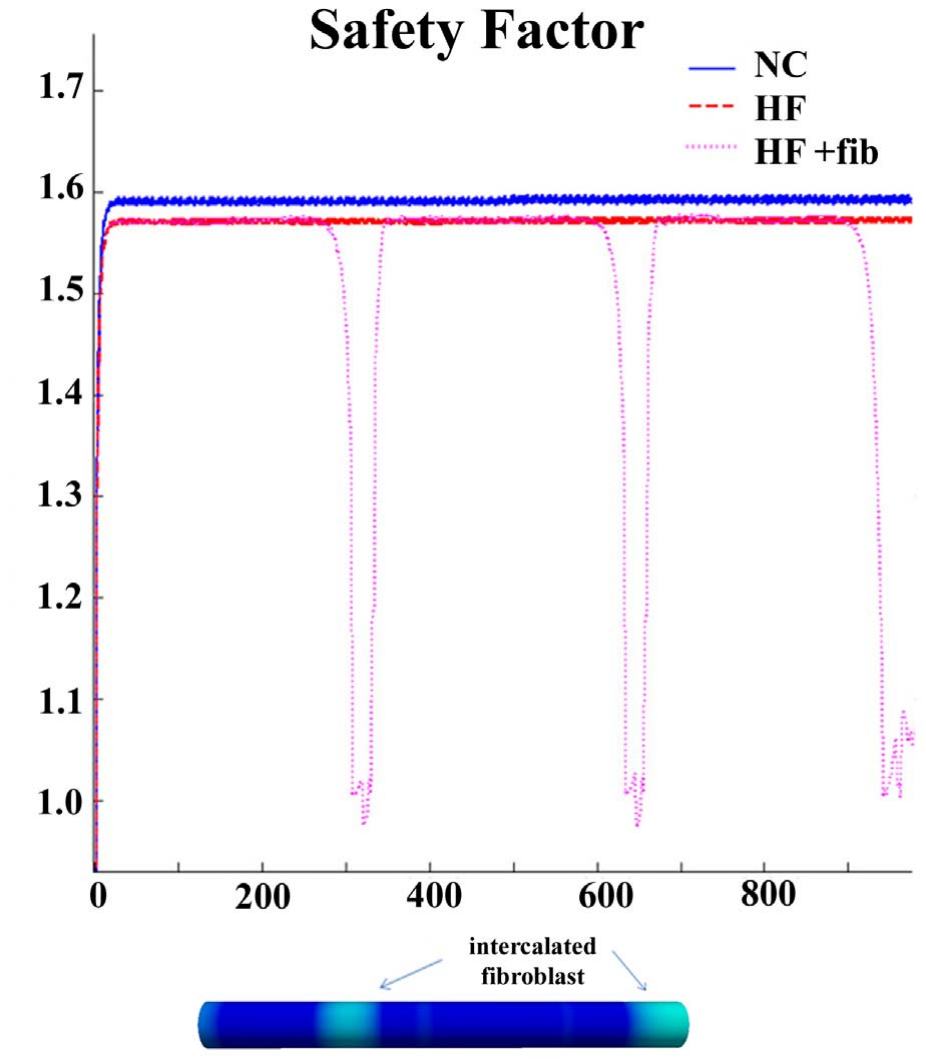
Safety factor for conduction in normal, failing conditions, and with fibrosis inserted. Safety factor (SF) calculated in a fiber of 900 endocardial cells under normal conditions (blue continuous line), when heart failure electrophysiological remodeling is considered (red dashed line), and when clustered fibrosis and concomitant heart failure electrophysiological remodeling are present (pink dotted line).

The safety in conduction was also evaluated, using the safety factor indicator (see Methods section). As show in Figure 8, the safety factor was very slightly reduced when HF electrophysiological remodeling was considered (red line) with respect to control conditions (blue line). However, when clustered fibrosis was introduced in the HF strand, safety factor was decreased yielding a value approx. 1 in the fibroblasts clusters, indicating that conduction is still possible but less safe. Thus, fibrosis slows CV and the quantity of charge that is able to be transmitted to the next cell is seriously compromised. When the fibrotic cluster was big enough, conduction was blocked.

## Discussion

### Major findings

This computational work, made possible by using two recent, comprehensive, and complimentary mathematical models of the human ventricular AP, provides important new insights into the role of HF-induced electrophysiological and structural remodeling in the modulation of transmural gradients for late repolarization, intracellular Ca^2+^ transient, and action potential conduction. The principal findings and insights from our work are (i) the demonstration that HF heterogeneous remodeling of I_NCX_ and I_SERCA_ modulates Ca^2+^ transient duration and the dispersion of AP-Ca^2+^ transient upstrokes delay. In addition, (ii) simulated homogeneous electrophysiological remodeling during HF increases APD dispersion (more significantly in ORd model than in GPB model), and (iii) HF structural remodeling enhances repolarization gradients and decreases CV and ERP. (iv) We suggest also that although M cells are known to contribute to the increase of APD and repolarization dispersion, they do not to alter the relative change from normal tissue to failing tissue; and (v) confirmation for the first time of the hypothesis that heterogeneous HF remodeling modulates repolarization gradients and can bring them below the values corresponding to normal conditions.

### Electrophysiological gradients in heart failure

Transmural heterogeneities of repolarization play a critical role in the genesis of polymorphic ventricular tachycardia (PVT) in HF [19]. Several experimental studies [19], [30], [31], [54] have addressed the impact of the dispersion of repolarization time in the transmural ventricular wall on the generation of reentrant activity in different animal species. In their study on dog ventricle, Akar et al. [19] demonstrated that spatial gradients of repolarization were significantly increased in HF because of the marked prolongation of APD in M cells and concluded that this was directly responsible for PVT generation. In the human right ventricle Lou et al. [30] also observed an increase in APD gradients in HF and related it to increased arrhythmogenesis. In contrast, Coronel et al. [60] observed that in the pig ventricle it is not only the repolarization gradient but also the restitution characteristics in combination with the time of arrival of the premature wavefront, which determines the occurrence of reentry.

Recent experiments have shown that TDR and APD gradient are reduced as a result of heart failure [31], [54]. These studies examined APD prolongation and dispersion of repolarization in coronary artery-perfused left ventricular wedge preparations from the human heart using optical mapping [31]. In these preparations APs were prolonged in HF in a transmurally heterogeneous manner, with the greatest prolongation occurring in the subepicardial layer, so that APD gradients from subendocardium to subepicardium were significantly decreased in failing compared with non-failing hearts. No significant M cell contribution could be identified in these HF wedges. Their identification of M cells was based on the local APD gradient between the areas of delayed repolarization (M cells) and the neighboring myocardium, taking into account intrinsic cellular properties and extrinsic coupling properties of the tissue. M cell islands were found in non-diseased hearts but this was not the case for the failing ones. Although M cells are present in the canine myocardium [19], [61], there is no evidence of the existence of this type of cells under failing conditions in human myocardium [30], [31], [54]. In the present study we analyzed the role of M cells and heterogeneous ionic remodeling in the modulation of repolarization gradients. Our results revealed increased APD dispersion under HF conditions assuming homogeneous remodeling with respect to control conditions. The presence of M cells increased general repolarization gradients but did not alter the relative change in HF with respect to control observed in the absence of M cells.

Assessing the importance of heterogeneous remodeling on the modulation of repolarization gradients was a major highlight in our simulations. Although changes in mRNA and protein expression across the transmural wall have been reported in failing human hearts [12]–[16], [20], much more experimental work is required to demonstrate how heterogeneous functional ionic remodeling can alter APD dispersion. Our theoretical results suggest that transmural heterogeneous remodeling of I_NCX_ and/or I_SERCA_ decreased APD dispersion with respect to homogeneous HF remodeling, regardless of the absence (Figure 3 red bars) or presence (Figure 3 green bars) of M cells. Furthermore, in the absence of M cells, dispersion of repolarization in the heterogeneously remodeled failing tissue, could be brought to lower values than in normal conditions. Thus, this is a plausible explanation for decreased APD dispersion and repolarization gradients in the experimental work of Glukhov et al. [21]. TDR was also modulated by heterogeneous remodeling and was decreased with respect to homogeneous remodeling when ORd model was used. In the presence of M cells TDR values were higher, and heterogeneous HF remodeling did not bring these values lower than in normal conditions as in [31], where M cells were absent.

The effects of heterogeneous remodeling on Ca^+2^ gradients were also analyzed. It is well known that myocytes in the failing heart exhibit abnormal intracellular Ca^+2^ transients, due in part to the decreased functional expression of the SR Ca^+2^ ATPase (SERCA2a), the enhanced sodium (Na^+^/Ca^+2^) exchanger function [62]–[64], and the aberrant SR Ca^+2^ leak, due to altered ryanodine receptor (RyR) function [65], [66]. Transmural differences in regulation of these Ca^2+^-cycling proteins were found in the failing ventricle [15], [16]. Our results accurately reproduce some of these features: the amplitude of Ca^+2^ transients decreases, and the rise and decay rates are slowed down, in accordance with experimental results [8], [67]. Furthermore, homogeneous HF remodeling increases CaTD_80_ dispersion, which is strongly modulated by heterogeneous remodeling of I_NCX_ and I_SERCA_. Experimentally, Lou et al. [54] found that the gradient of CaTD_80_ was lower in failing conditions. Our results can account for this only if heterogeneous remodeling (I_NCX_) and no M cells were considered in the analysis using ORd model. In our simulations AP-Ca delay dispersion was slightly increased (GPB) or unaltered (ORd), and heterogeneous remodeling lowered it below the values corresponding to the normal heart (ORd). Lou et al. [54] obtained a slightly lower gradient of AP-Ca delay in failing conditions than in normal conditions. Thus, heterogeneous remodeling could be a possible explanation for these results. Furthermore, heterogeneous remodeling of I_to_ [44], which was not considered in the present work, could also have a role in the modulation of Ca^2+^ transient waveform through changes in early repolarization [68].

### The role of structural remodeling in heart failure

In HF significant microanatomical remodeling related to arrhythmogenesis has been observed [69], [70]. The proliferation of fibroblasts and intercellular uncoupling during HF has been the focus of experimental [12], [50], [71]–[75] and simulation [29], [76], [77] studies. Experimental work has provided some insights into how gap junctions between myocytes are modified in the failing heart. Specifically, the proteins connexin 43 and 45 (Cx43, Cx45), are reduced and reorganized [21], [27], [50], [75], [78]. Furthermore, it has been hypothesized that cardiac fibroblasts and myocytes could be coupled to each other through gap junctions and modify electrical signal propagation actively, not only as passive insulators. Several experimental studies in vitro [24]–[28] corroborate this active interaction between both kinds of cells; however, experimental results in vivo with human cardiac tissue have not been reported. In a variety of species the degree of coupling between myocytes and fibroblasts has been analyzed [23], [29], [37], [52], [76], [79]–[81], and a wide range of values were found. In the absence of definitive data, modeling provides a powerful tool to explore repolarization abnormalities under such hypothetical conditions. Our simulation results showed that the presence of fibrosis significantly modified APD dispersion, TDR and other biomarkers (Figure 7). Inserted fibrosis and intercellular uncoupling increased TDR and APD dispersion, and a biphasic behavior was detected depending on the quantity of fibrotic content. This result might be related to the previous simulation studies reporting that intermediate fibrosis increases the vulnerability to reentry in a virtual infarcted rabbit heart [76] and in a virtual guinea pig fibrotic heart [77]. This is also illustrated in our accompanying paper [82] in the setting of simulated human heart failure. Excitability was also altered in the presence of fibroblasts through the elevation of the resting potential as reported before [37], [80], [83]. Thus, structural remodeling modified the electrical properties of the cardiac tissue generating a substrate for arrhythmogenesis [28], [76], [77], [84], as we illustrated in our accompanying paper [82]. A local decrease in CV when fibrosis was inserted concomitant with intercellular uncoupling was found in our simulations and is in agreement with experimental results [21], [24], [25], [84]–[86]. In addition, the ERP was enhanced by ionic remodeling present in HF in agreement with experimental results [21] but was decreased as the fibrotic content increased. This effect may favor the vulnerability to reentry [87], as was reported in other simulation studies [76], [77]. In this study we also demonstrated for the first time that the safety factor for conduction was reduced in the presence of fibrotic clusters, as stated in Glukhov et al. [21], and propagation block could be elicited.

### Limitations of the study

Several limitations need to be considered, when drawing inference from mathematical modeling. Our myocyte model for HF based on changes in the ion channel parameters has the inherited limitations described in [41]. Mainly, data from a large number of experimental studies were taken into account, thus resulting in a high variability not only in the ionic remodeling but also in the stage and etiology of HF and its phenotype. For this reason, a sensitivity analysis was performed in our previous work [42], where the experimental variability in ionic remodeling was taken into account and yielded values of APD_90_ and other HF biomarkers within experimental observations. In addition, our HF model was implemented employing two different human AP models: GPB and ORd. These two models were built and validated on the basis of extensive human data. However, there are important discrepancies in their formulations of K^+^ currents and Ca^2+^ handling, leading to differences in the simulation results regarding repolarization and Ca^2+^ transient behavior. Thus, caution should be exercised when comparing simulation results. With regard to heterogeneous remodeling, only the changes in I_NCX_ and I_SERCA_ were applied heterogeneously within the ventricular wall, on the basis of the limited human data available. Experimental results carried out in human hearts have also reported a transmural heterogeneous remodeling in K^+^ currents [20], [44]. Although transmural differences in the remodeling of transient outward K^+^ current would have affect mainly early repolarization, which was out of the scope of the present analysis, they might also have an impact on Ca^2+^ transient waveform and its dispersion within the ventricular wall [68]. Further combinations of heterogeneous remodeling will be considered in our future work. Finally, to simulate the interaction between human ventricular myocytes and fibroblasts we used an ionic fibroblast model [37] based on experimental data from adult rat fibroblasts, as no voltage clamp data or ionic models of human ventricular fibroblasts have been published so far. Possible inter-species differences might affect the results of our simulations.

### Clinical implications and conclusions

This study aimed to investigate *in silico* the effects of microanatomical and electrophysiological remodeling on the functional properties (phenotype) of the human failing ventricle. Two of the most recent and detailed human AP models (GPB and ORd) were modified to mimic HF phenotype.

Transmurally heterogeneous HF induced ionic remodeling modulated repolarization and Ca^2+^ transient gradients, which could be driven to values lower than the gradients corresponding to normal tissue. In contrast, homogeneous HF ionic remodeling, intercellular uncoupling, and fibrosis increased these gradients. Structural remodeling also altered conduction properties setting the substrate for arrhythmogenesis. Our findings may have important consequences for the treatment and prevention of human HF-induced arrhythmias and its potential contribution to mortality. Pharmacological modulation of intercellular coupling with rotigaptide, an antiarrhythmic substance increasing gap junctional conductance, may have beneficial effects in patients with heart failure. The concomitant reduction of the amount of fibrosis might be successful in these patients but requires further investigation.

## Supporting Information

File S1 Figure S1, Random configurations for 10% fibrosis in the multicellular strand. The multicellular strand is composed by 165 nodes, where the fibroblast model is solved in 10% of the nodes (light color) and the myocyte AP model is solved in the rest of the nodes (blue). See Methods for details. Figure S2, Random configurations for 20% fibrosis in the multicellular strand. The multicellular strand is composed by 165 nodes, where the fibroblast model is solved in 20% of the nodes (light color) and the myocyte AP model is solved in the rest of the nodes (blue). See Methods for details. Figure S3, APD dispersion for normal coupling. Boxplots showing action potential duration (ADP) dispersion for low fibrosis (10%) and high fibrosis (20%), and D_M_ = 0.0006 cm/ms. Figure S4, TDR for normal coupling. Boxplots showing transmural dispersion (TDR) for low fibrosis (10%) and high fibrosis (20%), and D_M_ = 0.0006 cm/ms. Figure S5, APD dispersion for severe uncoupling. Boxplots showing action potential duration (ADP) dispersion for low fibrosis (10%) and high fibrosis (20%), and D_M_ = 0.00025 cm/ms. Figure S6, TDR for severe uncoupling. Boxplots showing transmural dispersion (TDR) for low fibrosis (10%) and high fibrosis (20%), and D_M_ = 0.00025 cm/ms. Figure S7, APD dispersion for intermediate uncoupling. Boxplots showing action potential duration (ADP) dispersion for low fibrosis (10%) and high fibrosis (20%), and D_M_ = 0.0003 cm/ms. Figure S8, TDR for intermediate uncoupling. Boxplots showing transmural dispersion (TDR) for low fibrosis (10%) and high fibrosis (20%), and D_M_ = 0.0003 cm/ms. Figure S9, APD dispersion for mild uncoupling. Boxplots showing action potential duration (ADP) dispersion for low fibrosis (10%) and high fibrosis (20%), and D_M_ = 0.00045 cm/ms. Figure S10, TDR for mild uncoupling. Boxplots showing transmural dispersion (TDR) for low fibrosis (10%) and high fibrosis (20%), and D_M_ = 0.00045 cm/ms. Table S1, APD dispersion and TDR for 10% fibrosis. Action potential duration (APD) dispersion and transmural dispersion (TDR) for the random configurations corresponding to a fibrotic content of 10% and normal intercellular coupling (D_M_ = 0.0006 cm/ms). Table S2, APD dispersion and TDR for 20% fibrosis. Action potential duration (APD) dispersion and transmural dispersion (TDR) for the random configurations corresponding to a fibrotic content of 20% and normal intercellular coupling (D_M_ = 0.0006 cm/ms). Table S3, APD dispersion for 10% fibrosis and intercellular uncoupling. Action potential duration (APD) dispersion for the random configurations corresponding to a fibrotic content of 10% and several degrees of intercellular uncoupling (D_M_ in cm/ms). Table S4, TDR for 10% fibrosis and intercellular uncoupling. Transmural dispersion of repolarization (TDR) for the random configurations corresponding to a fibrotic content of 10% and several degrees of intercellular uncoupling (D_M_ in cm/ms). Table S5, APD dispersion for 20% fibrosis and intercellular uncoupling Action potential duration (APD) dispersion for the random configurations corresponding to a fibrotic content of 20% and several degrees of intercellular uncoupling (D_M_ in cm/ms). Table S6, TDR for 20% fibrosis and intercellular uncoupling. Transmural dispersion of repolarization (TDR) for the random configurations corresponding to a fibrotic content of 20% and several degrees of intercellular uncoupling (D_M_ in cm/ms).
(DOCX)
