# Supplementary material for: Electrophysiological and Structural Remodeling in Heart Failure Modulate Arrhythmogenesis. 1D Simulation Study
Source: PLoS One. 2014 Sep 5;9(9):e106602. doi: 10.1371/journal.pone.0106602 (PMC4156355; doi:10.1371/journal.pone.0106602)
Supplement: File S1 — Figure S1, Random configurations for 10% fibrosis in the multicellular strand. The multicellular strand is composed by 165 nodes, where the fibroblast model is solved in 10% of the nodes (light color) and the myocyte AP model is solved in the rest of the nodes (blue). See Methods for details. Figure S2, Random configurations for 20% fibrosis in the multicellular strand. The multicellular strand is composed by 165 nodes, where the fibroblast model is solved in 20% of the nodes (light color) and the myocyte AP model is solved in the rest of the nodes (blue). See Methods for details. Figure S3, APD dispersion for normal coupling. Boxplots showing action potential duration (ADP) dispersion for low fibrosis (10%) and high fibrosis (20%), and DM = 0.0006 cm/ms. Figure S4, TDR for normal coupling. Boxplots showing transmural dispersion (TDR) for low fibrosis (10%) and high fibrosis (20%), and DM = 0.0006 cm/ms. Figure S5, APD dispersion for severe uncoupling. Boxplots showing action potential duration (ADP) dispersion for low fibrosis (10%) and high fibrosis (20%), and DM = 0.00025 cm/ms. Figure S6, TDR for severe uncoupling. Boxplots showing transmural dispersion (TDR) for low fibrosis (10%) and high fibrosis (20%), and DM = 0.00025 cm/ms. Figure S7, APD dispersion for intermediate uncoupling. Boxplots showing action potential duration (ADP) dispersion for low fibrosis (10%) and high fibrosis (20%), and DM = 0.0003 cm/ms. Figure S8, TDR for intermediate uncoupling. Boxplots showing transmural dispersion (TDR) for low fibrosis (10%) and high fibrosis (20%), and DM = 0.0003 cm/ms. Figure S9, APD dispersion for mild uncoupling. Boxplots showing action potential duration (ADP) dispersion for low fibrosis (10%) and high fibrosis (20%), and DM = 0.00045 cm/ms. Figure S10, TDR for mild uncoupling. Boxplots showing transmural dispersion (TDR) for low fibrosis (10%) and high fibrosis (20%), and DM = 0.00045 cm/ms. Table S1, APD dispersion and TDR for 10% fibrosis. Action potential [file pone.0106602.s001.docx]

**SUPPORTING INFORMATION S1**

*RANDOM FIBROSIS CONFIGURATIONS*

The electrical activity of multicellular strands of 165 nodes was simulated containing coupled myocytes and fibroblasts. Fibroblasts distribution was organized randomly, by assigning a probabilistic function. Fibrotic contents of 10% and 20% were chosen corresponding to the percentage of nodes assigned to the fibroblast ionic model. For each fibrotic content 11 random configurations were simulated and are shown in Figure S1 (fibrotic content of 10%) and Figure S2 (fibrotic content of 20%). These gave rise to different levels of fibrosis (represented in light blue in the figures) quantified as the percentage of nodes executing the fibroblast model (P_f_) comprised between 9.03% and 10.84% (15 and 18 nodes, respectively), for a fibrotic content of 10% and P_f_ comprised between 19.87% and 22.89% (33 and 38 nodes, respectively) for a fibrotic content of 20%.

The dispersion of action potential duration (APD) and transmural dispersion of repolarization (TDR) were calculated (as described in the methods section of the main text) in all the configurations. Tables S1 and S2 show the results for a fibrotic content of 10% and 20%, respectively, and a normal intercellular coupling (D_M_=0.0006 cm/ms). The results of configuration 1 are shown in the main text and Figures of the article.

A statistical analysis was performed to evaluate the mean and the standard deviation. Values are expressed as mean±SD in the last line of Tables S1 and S2.

To evaluate the effects of the different degrees of fibrosis (10% and 20%) on APD dispersion and TDR, boxplots diagrams for AP dispersion (Figure S3) and TDR (Figure S4) for a fibrotic content of 10% and 20% were performed with SPSS Statistics 17.0 software (IBM SPSS Statistics). The difference of means using student t-test was also evaluated and p<0.05 was considered significant. In the case of APD dispersion, the difference between 10% and 20% fibrosis was considered significative. For TDR, although the mean for 10% of fibrosis was higher than for 20%, the difference was not significative.

The same analyses were performed for different intercellular coupling degrees and the results are shown in tables S3, S4, S5 and S6. The corresponding boxplots are depicted in Figures S5, S6, S7, S8, S9 and S10. For the different intercellular coupling degrees 10% fibrosis presents always bigger APD dispersion and TDR than for 20%.


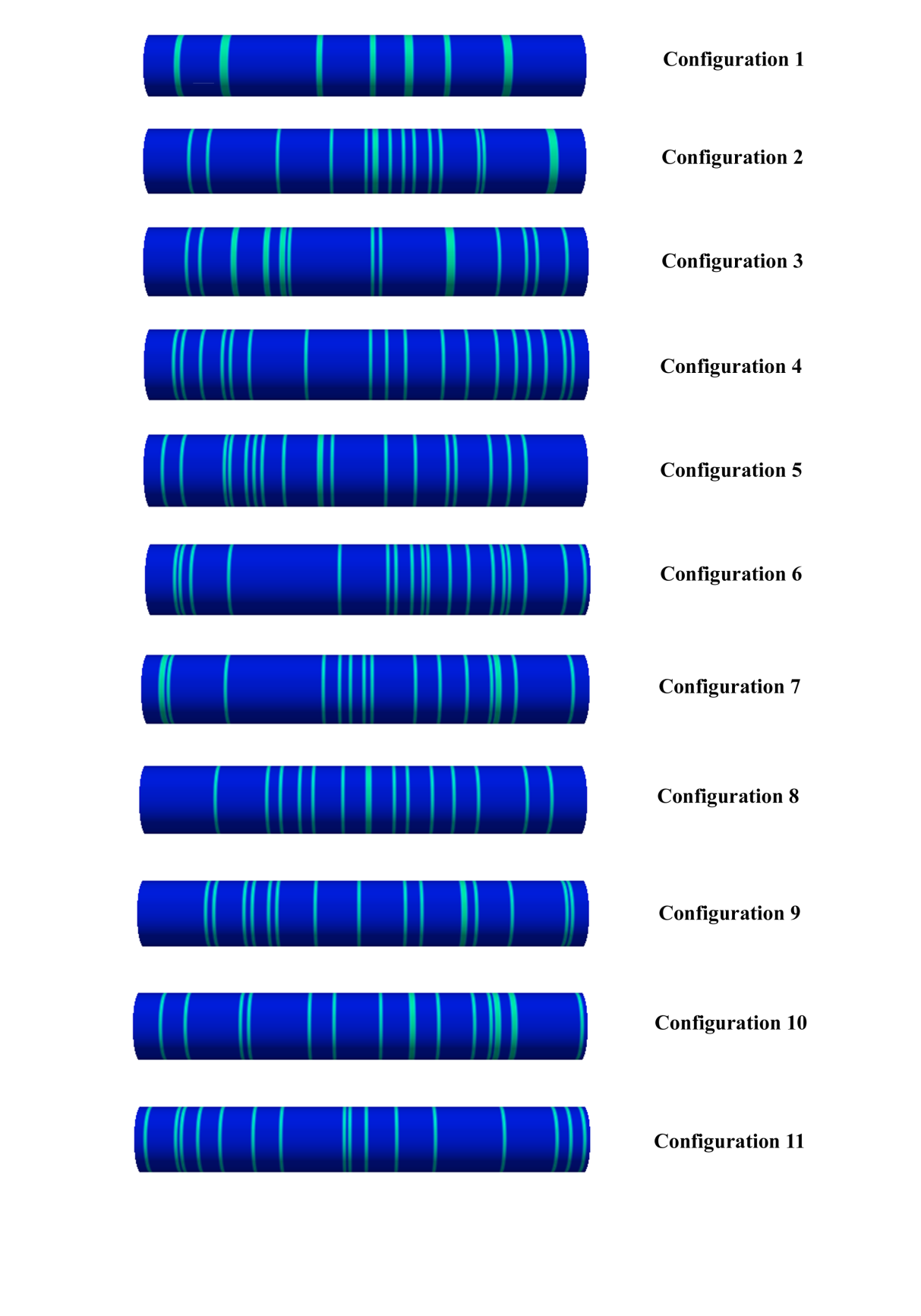


**Figure S1.** **Random configurations for 10 % fibrosis in the multicellular strand.** The multicellular strand is composed by 165 nodes, where the fibroblast model is solved in 10% of the nodes (light color) and the myocyte AP model is solved in the rest of the nodes (blue). See Methods for details.


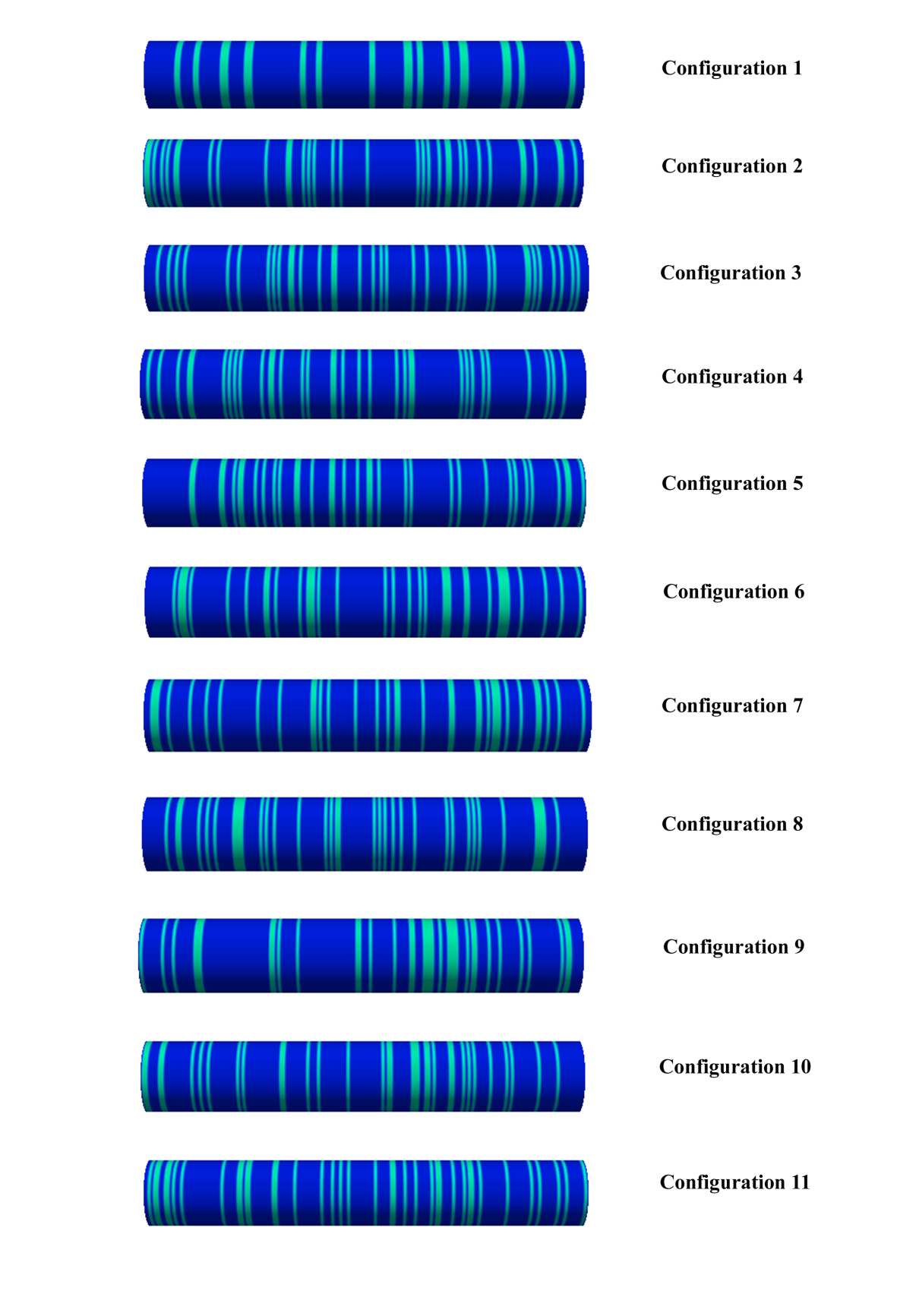


**Figure S2.** **Random configurations for 20 % fibrosis in the multicellular strand.** The multicellular strand is composed by 165 nodes, where the fibroblast model is solved in 20% of the nodes (light color) and the myocyte AP model is solved in the rest of the nodes (blue). See Methods for details.

**Table S1.** **APD dispersion and TDR for 10% fibrosis.** Action potential duration (APD) dispersion and transmural dispersion (TDR) for the random configurations corresponding to a fibrotic content of 10% and normal intercellular coupling (D_M_=0.0006 cm/ms).

| **Configurations** | **APD dispersion (ms)** | **TDR (ms)** |
| --- | --- | --- |
| Config. 1 | 70 | 97 |
| Config. 2 | 88 | 59 |
| Config. 3 | 38 | 53 |
| Config. 4 | 57 | 50 |
| Config. 5 | 71 | 106 |
| Config. 6 | 92 | 71 |
| Config. 7 | 60 | 38 |
| Config. 8 | 112 | 88 |
| Config. 9 | 59 | 48 |
| Config. 10 | 44 | 33 |
| Config. 11 | 46 | 76 |
|  | | |
| ***Mean ± SD*** | 67±23 | 97±60 |

**Table S2.** **APD dispersion and TDR for 20% fibrosis.** Action potential duration (APD) dispersion and transmural dispersion (TDR) for the random configurations corresponding to a fibrotic content of 20% and normal intercellular coupling (D_M_=0.0006 cm/ms).

| **Configurations** | **APD dispersion (ms)** | **TDR (1ms)** |
| --- | --- | --- |
| Config. 1 | 24 | 64 |
| Config. 2 | 30 | 37 |
| Config. 3 | 28 | 37 |
| Config. 4 | 28 | 75 |
| Config. 5 | 80 | 65 |
| Config. 6 | 31 | 45 |
| Config. 7 | 35 | 40 |
| Config. 8 | 25 | 78 |
| Config. 9 | 48 | 44 |
| Config. 10 | 44 | 88 |
| Config. 11 | 24 | 48 |
|  | | |
| ***Mean ± SD*** | 36±16 | 56±18 |


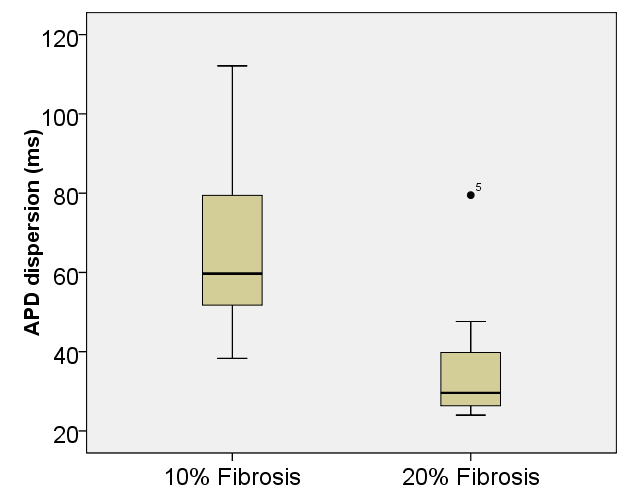


**Figure S3.** **APD dispersion for normal coupling.** Boxplots showing action potential duration (ADP) dispersion for low fibrosis (10%) and high fibrosis (20%), and D_M_=0.0006 cm/ms.


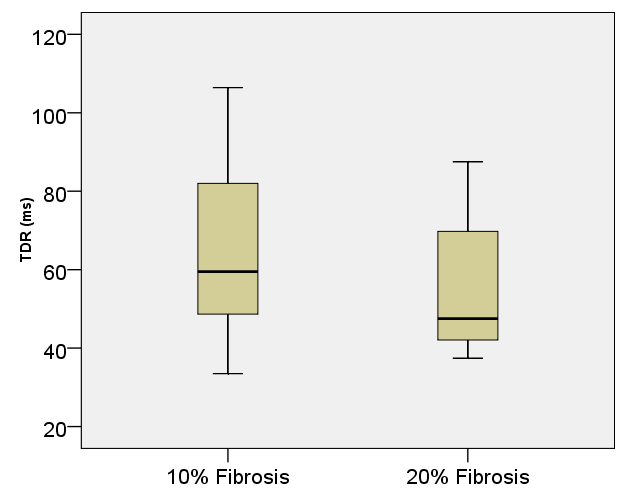


**Figure S4. TDR for normal coupling.** Boxplots showing transmural dispersion (TDR) for low fibrosis (10%) and high fibrosis (20%), and D_M_=0.0006 cm/ms.

**Table S3.** **APD dispersion for 10% fibrosis and intercellular uncoupling.** Action potential duration (APD) dispersion for the random configurations corresponding to a fibrotic content of 10% and several degrees of intercellular uncoupling (D_M_ in cm/ms).

| **Configurations**  **(HF 10% Fib)** | **D_M_ =0.00025** | **D_M_ =0.0003** | **D_M_ =0.00045** |
| --- | --- | --- | --- |
|  | **APD dispersion (ms)** | | |
| Config.1 | 99 | 92 | 79 |
| Config. 2 | 109 | 104 | 94 |
| Config. 3 | 67 | 61 | 47 |
| Config. 4 | 74 | 70 | 62 |
| Config. 5 | 94 | 89 | 78 |
| Config. 6 | 120 | 113 | 100 |
| Config. 7 | 84 | 77 | 66 |
| Config. 8 | 141 | 135 | 121 |
| Config. 9 | 92 | 84 | 67 |
| Config. 10 | 52 | 49 | 46 |
| Config. 11 | 65 | 61 | 52 |
|  |  |  |  |
| ***Mean ± SD*** | 90±26 | 85±25 | 73±23 |

**Table S4.** **TDR for 10% fibrosis and intercellular uncoupling.** Transmural dispersion of repolarization (TDR) for the random configurations corresponding to a fibrotic content of 10% and several degrees of intercellular uncoupling (D_M_ in cm/ms).

| **Configurations**  **(HF 10% Fib)** | **D_M_ =0.00025** | **D_M_ =0.0003** | **D_M_ =0.00045** |
| --- | --- | --- | --- |
|  | **TDR (ms)** | | |
| Config. 1 | 128 | 120 | 106 |
| Config. 2 | 80 | 73 | 65 |
| Config. 3 | 83 | 76 | 61 |
| Config. 4 | 72 | 66 | 54 |
| Config. 5 | 136 | 129 | 115 |
| Config. 6 | 97 | 91 | 78 |
| Config. 7 | 56 | 52 | 43 |
| Config. 8 | 114 | 108 | 96 |
| Config. 9 | 82 | 74 | 58 |
| Config. 10 | 49 | 45 | 38 |
| Config. 11 | 103 | 97 | 83 |
|  |  |  |  |
| ***Mean ± SD*** | 90±27 | 84±26 | 72±25 |

**Table S5.** **APD dispersion for 20% fibrosis and intercellular uncoupling** Action potential duration (APD) dispersion for the random configurations corresponding to a fibrotic content of 20% and several degrees of intercellular uncoupling (D_M_ in cm/ms).

| **Configurations**  **(HF 20% Fib)** | **D_M_ =0.00025** | **D_M_ =0.0003** | **D_M_ =0.00045** |
| --- | --- | --- | --- |
|  | **APD dispersion (ms)** | | |
| Config. 1 | 37 | 34 | 28 |
| Config. 2 | 38 | 36 | 32 |
| Config. 3 | 33 | 32 | 29 |
| Config. 4 | 35 | 33 | 30 |
| Config. 5 | 102 | 97 | 86 |
| Config. 6 | 43 | 40 | 34 |
| Config. 7 | 45 | 42 | 38 |
| Config. 8 | 36 | 33 | 28 |
| Config. 9 | 70 | 64 | 54 |
| Config. 10 | 54 | 52 | 47 |
| Config. 11 | 32 | 30 | 26 |
|  |  |  |  |
| ***Mean ± SD*** | 47±21 | 44±20 | 39±17 |

**Table S6. TDR for 20% fibrosis and intercellular uncoupling**. Transmural dispersion of repolarization (TDR) for the random configurations corresponding to a fibrotic content of 20% and several degrees of intercellular uncoupling (D_M_ in cm/ms).

| **Configurations**  **(HF 20% Fib)** | **D_M_ =0.00025** | **D_M_ =0.0003** | **D_M_ =0.00045** |
| --- | --- | --- | --- |
|  | **TDR (ms)** | | |
| Config. 1 | 37 | 34 | 28 |
| Config. 2 | 45 | 42 | 38 |
| Config. 3 | 44 | 42 | 39 |
| Config. 4 | 79 | 79 | 73 |
| Config. 5 | 88 | 82 | 71 |
| Config. 6 | 53 | 51 | 47 |
| Config. 7 | 44 | 42 | 42 |
| Config. 8 | 93 | 90 | 83 |
| Config. 9 | 51 | 48 | 44 |
| Config. 10 | 95 | 93 | 89 |
| Config. 11 | 46 | 45 | 42 |
|  |  |  |  |
| ***Mean ± SD*** | 61±22 | 58±22 | 54±20 |


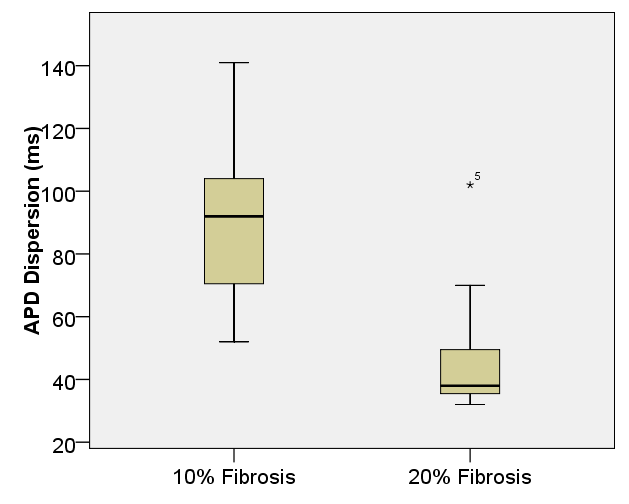


**Figure S5.** **APD dispersion for severe uncoupling.**Boxplots showing action potential duration (ADP) dispersion for low fibrosis (10%) and high fibrosis (20%), and D_M_=0.00025 cm/ms.


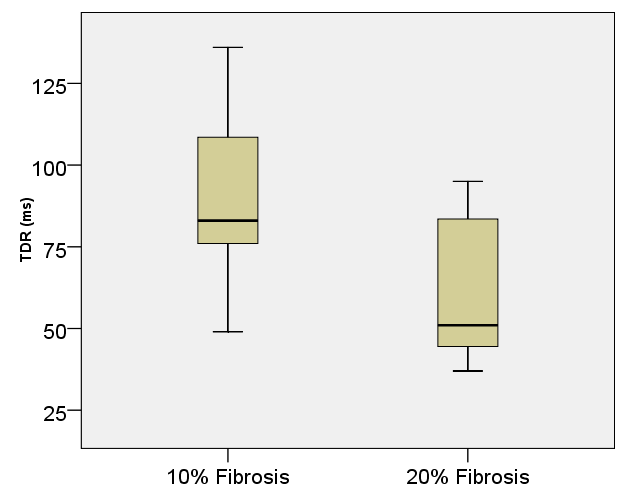


**Figure S6. TDR for severe uncoupling.** Boxplots showing transmural dispersion (TDR) for low fibrosis (10%) and high fibrosis (20%), and D_M_=0.00025 cm/ms.


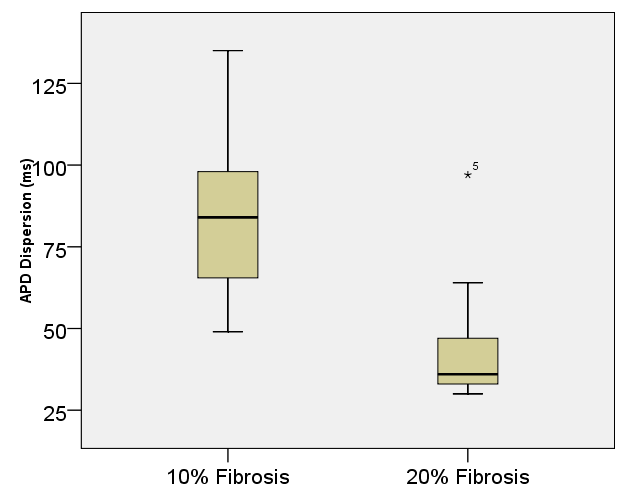


**Figure S7.** **APD dispersion for intermediate uncoupling.** Boxplots showing action potential duration (ADP) dispersion for low fibrosis (10%) and high fibrosis (20%), and D_M_=0.0003 cm/ms.


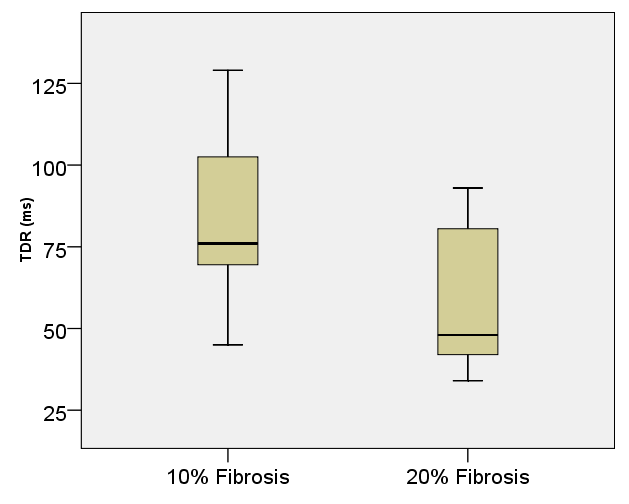


**Figure S8. TDR for intermediate uncoupling.** Boxplots showing transmural dispersion (TDR) for low fibrosis (10%) and high fibrosis (20%), and D_M_=0.0003 cm/ms.


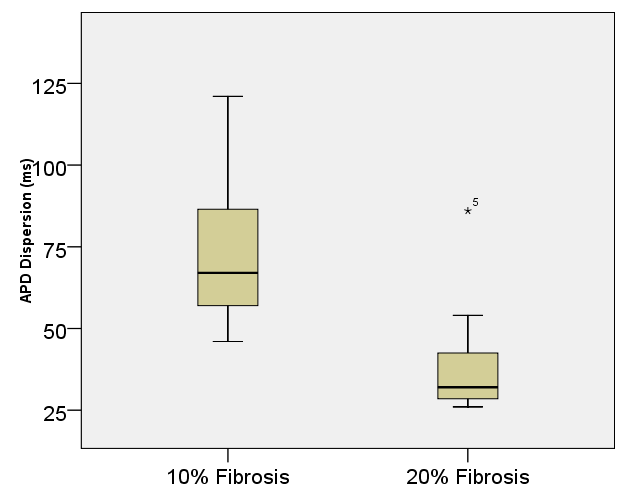


**Figure S9.** **APD dispersion for mild uncoupling.** Boxplots showing action potential duration (ADP) dispersion for low fibrosis (10%) and high fibrosis (20%), and D_M_=0.00045 cm/ms.


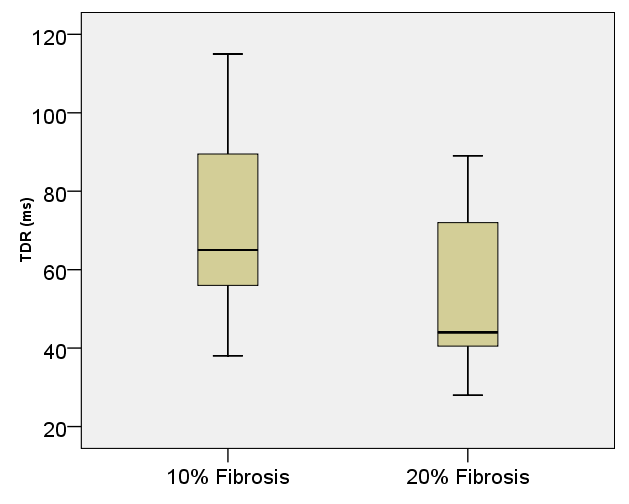


**Figure S10. TDR for mild uncoupling.** Boxplots showing transmural dispersion (TDR) for low fibrosis (10%) and high fibrosis (20%), and D_M_=0.00045 cm/ms.
